# Serotonin is elevated in risk-genotype carriers of TCF7L2 - rs7903146

**DOI:** 10.1038/s41598-019-49347-y

**Published:** 2019-09-06

**Authors:** Andreas Leiherer, Axel Muendlein, Christoph H. Saely, Peter Fraunberger, Heinz Drexel

**Affiliations:** 1 Vorarlberg Institute for Vascular Investigation and Treatment (VIVIT), Feldkirch, Austria; 2 Department of Medicine and Cardiology, Academic Teaching Hospital Feldkirch, Feldkirch, Austria; 3 Private University of the Principality of Liechtenstein, Triesen, Liechtenstein; 4 Medical Central Laboratories, Feldkirch, Austria; 5 Drexel University College of Medicine, Philadelphia, PA USA; 6 Division of Angiology, Swiss Cardiovascular Center, University Hospital of Bern, Bern, Switzerland

**Keywords:** Metabolomics, Transcription factors, Genomics, Type 2 diabetes, Risk factors

## Abstract

The transcription factor 7-like 2 (TCF7L2) polymorphism rs7903146 is known to be tightly associated with an elevated risk for type 2 diabetes, whereas the molecular mechanisms remain elusive. We evaluated the metabolic profile of a total of 394 patients’ serum samples with respect to their rs7903146 genotype using targeted metabolomics in a discovery (n = 154) and a validation (n = 240) study. We have identified serotonin as the top metabolite being increased in carriers of the risk allele. Serotonin was significantly associated with the rs7903146 genotype after full adjustment including type 2 diabetes and further top ranked metabolites. Given the role of peripheral serotonin in metabolic homeostasis and type 2 diabetes, this finding provides a first hint that the well-known impact of the TCF7L2 polymorphism on type 2 diabetes risk may involve a serotonin-dependent pathway.

## Introduction

Development of type 2 diabetes is linked with dysregulated metabolism. The role of metabolic homeostasis and cellular sensors has recently drawn attention to peripheral serotonin which is elevated in diabetic subjects^1,2^. Although strategies interfering with serotonin synthesis are believed to hold great potential to treat type 2 diabetes in humans, serotonin signalling in the periphery is immensely complex and still poorly understood^1,2^.

Genetic background analyses of type 2 diabetes in populations of different ethnic descent reproducibly identified single nucleotide polymorphisms (SNPs) of transcription factor 7-like 2 (TCF7L2) as one of the most important loci to predispose carriers for type 2 diabetes^3–5^. Among said SNPs, variant rs7903146 showed the most significant association with type 2 diabetes risk^3,6–9^. TCF7L2, also known as TCF4, is located on chromosome 10q25.3^10^ and encodes a transcription factor that serves as a nuclear receptor for β-catenin in the Wingless-type (Wnt) signalling pathway^11^. Several studies have shown that TCF7L2 influences the risk of type 2 diabetes via impairment of β-cell function (BCF)^4,8^, impacting both, glucose tolerance and insulin secretion^12^. However, the exact mechanisms through which TCF7L2 and respective polymorphisms alter metabolic processes and affect the susceptibility to type 2 diabetes remain to be established^13,14^.

Integrated metabolomics and genomics hold great potential for uncovering the association of an individual metabolite with a single genotype and allow to generate hypotheses for mechanisms underlying the effect of a specific SNP or gene^15^. A previous metabolomics profiling of 41 KORA study participants carried out in conjunction with a glucose tolerance test revealed alterations in phospholipid metabolism in individuals with the risk TCF7L2 genotype. To the contrary, a non-targeted metabolomics profiling of 30 TUEF study participants revealed no difference between homozygous carriers and non-carriers of the risk allele^16^.

In view of this inconsistency, we analysed the metabolic alterations in a much larger patient population, comprising of a discovery and a validation cohort with a total of 394 patients.

## Results

### Patient characteristics and subgroup analysis

In the discovery study, we randomly selected 154 from a total of 1660 genotyped patients including 84 subjects with the risk allele (CT/TT) and 70 carrying no risk allele (CC). In the validation study, we randomly selected 240 patients with 105 carrying the risk allele and 135 patients without the risk allele. In total, 394 patients carrying either the CT/TT (n = 189) or the CC (n = 205) allele combination were enrolled for metabolomics studies. Table 1 presents patient characteristics for both study populations with respect to TCF7L2 rs7903146.

**Table 1:** Patient characteristics.

	Discovery study			Validation study		
	CC genotype	CT/TT genotype	p	CC genotype	CT/TT genotype	p
	mean ± SD	mean ± SD		mean ± SD	mean ± SD	
Age (years)	65.93 ± 9.71	63.60 ± 9.31	0.091	65.34 ± 8.39	66.49 ± 8.43	0.255
Male sex (%)	52.9	46.4	0.427	47.6	54.8	0.268
BMI (kg/m^2^)	28.60 ± 5.25	28.32 ± 5.30	0.558	29.64 ± 4.79	28.78 ± 3.81	0.164
Waist circumference (cm)	102 ± 13	100 ± 13	0.232	103 ± 11	103 ± 11	0.860
Hypertension (%)	70.0	66.7	0.658	78.1	79.3	0.827
Triglycerides (mg/dl(mmol/l))	129(1.5) ± 57(0.6)	144(1.6) ± 106(1.2)	0.755	170(1.9) ± 125(1.4)	151(1.7) ± 76(0.9)	0.674
LDL-C (mg/dl(mmol/l))	133(3.44) ± 44(1.14)	126(3.26) ± 37(0.96)	0.306	129(3.34) ± 45(1.17)	127(3.29) ± 39(1.01)	0.878
HDL-C (mg/dl(mmol/l))	56(1.45) ± 17(0.44)	59(1.53) ± 20(0.52)	0.291	53(1.37) ± 16(0.41)	55(1.42) ± 19(0.49)	0.645
CAD, significant (%)	48.6	47.6	0.906	45.7	58.5	0.049
Type 2 diabetes (%)	24.3	27.4	0.663	29.5	40.0	0.092
Alcohol consumption (%)	57.9	61.0	0.714	57.4	52.4	0.448
Smoking, current (%)	11.4	25.0	0.032	13.3	16.3	0.524
Glucose, fasting (mg/dl(mmol/l))	105 (5.8) ± 202 (1.1)	116 (6.4) ± 40 (2.2)	0.236	117 (6.5) ± 46 (2.5)	122 (6.7) ± 45 (2.5)	0.087
Glucose, 2 h OGTT (mg/dl)	123 (6.8) ± 47 (2.6)	126 (7.0) ± 63 (3.5)	0.704	151 (8.4) ± 83 (4.6)	153 (8.5) ± 80 (4.4)	0.826
HbA1c (% (mmol/mol))	5.88 (41) ± 0.57 (6)	6.18 (44) ± 0.97 (11)	0.260	6.22 (44) ± 1.21 (13)	6.43 (47) ± 1.25 (14)	0.026
Insulin (µU/ml)	12.73 ± 9.13	12.85 ± 11.19	0.676	13.96 ± 13.84	21.52 ± 77.42	0.299
eGFR (ml/min/1.73 m^2^)	96.37 ± 16.23	95.94 ± 17.89	0.862	94.12 ± 14.56	93.96 ± 17.48	0.604
ASA treatment (%)	70.0	73.8	0.600	73.3	62.2	0.069
Beta blocker treat. (%)	55.7	44.0	0.149	57.1	54.8	0.719
ACE inhibitor treat. (%)	40.0	27.4	0.098	28.6	31.1	0.670
ARB treat. (%)	10.0	8.3	0.720	11.4	10.4	0.794
Statin treatment (%)	35.7	46.4	0.179	51.4	48.1	0.614
Fibrate treat. (%)	0.0	1.2	0.360	2.9	3.7	0.717
Sulfonylurea treat. (%)	2.9	4.8	0.543	8.6	10.4	0.639
Biguanide treat. (%)	10.0	11.9	0.707	10.5	12.6	0.612
Glitazone treat. (%)	1.4	1.2	0.897	0.0	0.0	n.a.
Insulin treat. (%)	5.7	7.1	0.720	5.7	8.1	0.466
Antidiabetic treat. (%)	17.1	16.7	0.937	17.1	19.3	0.674

The table presents the patient characteristics of the discovery study and of the validation study. Patients were separated with respect to their genotype. Data are given as means ± standard deviations as indicated. Hba1c is given according to DCCT and IFCC -derived units (%; mmol/mol). ACE denotes angiotensin converting enzyme, ARB angiotensin II receptor blocker, ASA acetyl salicylic acid, BMI body mass index, CAD coronary artery disease, which is defined by an angiographically determined coronary artery stenosis with lumen narrowing ≥50%, eGFR the estimated glomerular filtration rate, HbA1c hemoglobin A1c, HDL-C high density lipoprotein cholesterol, and LDL-C low density lipoprotein cholesterol.

### Metabolomic profiling

**Figure 1:**
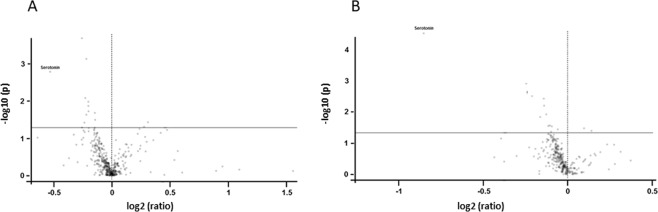
Volcano plot. The volcano plot depicts the combination of metabolite ratios of wildtype and risk allele carriers (ratio) and t-test p-values (p) in the discovery study (**A**) and the validation study (**B**). The x-axis represents log2 (ratio), the Y-axis is −log10 (p), based on raw p-values. The black horizontal line represents threshold for p-value (0.05).

Metabolite concentrations in the discovery study samples (n = 154) were analysed with respect to TCF7L2 rs79303146 genotype. We then compared the metabolite ratios between the CC and the CT/TT group. The results are summarized in Table 2 and Fig. 1. The biogenic amine serotonin was identified as the metabolite with the lowest ratio (0.69; p-value = 1.67 E-3), representing a 31% lower concentration of serotonin in the CC-group compared to the CT/TT group. This is followed by the ceramide N-C12:0-Cer and several phosphatidylcholines (PC).

**Table 2:** Metabolites significantly associated with the TCF7L2 rs7903146 genotype.

Study	Metabolite	Class	ratio	abs ratio	log2(ratio)	p	−log10(p)
Discovery	Serotonin	biogenic amine	0.693	30.7%	−0.530	1.67 E-3	2.777
Discovery	N-C12:0-Cer	ceramide	1.241	24.1%	0.311	0.037	1.426
Discovery	LPC a C18:0	lysophosphatidylcholin (acyl bond)	1.212	21.2%	0.278	0.049	1.307
Discovery	PC aa C36:6	phosphatidylcholin (diacyl bond)	0.849	15.1%	−0.236	0.038	1.418
Discovery	PC aa C38:6	phosphatidylcholin (diacyl bond)	0.854	14.6%	−0.227	0.008	2.075
Discovery	H1	hexoses	0.859	14.1%	−0.219	7.57 E-4	3.121
Discovery	PC aa C40:6	phosphatidylcholin (diacyl bond)	0.860	14.0%	−0.218	0.024	1.628
Discovery	C3-OH	Acylcarnitine	0.870	13.0%	−0.201	0.011	1.975
Discovery	PC aa C34:4	phosphatidylcholin (diacyl bond)	0.870	13.0%	−0.201	0.014	1.863
Discovery	C6:1	Acylcarnitine	0.871	12.9%	−0.200	0.019	1.720
Discovery	PC aa C36:4	phosphatidylcholin (diacyl bond)	0.904	9.6%	−0.146	0.021	1.677
Discovery	PC aa C38:4	phosphatidylcholin (diacyl bond)	0.906	9.4%	−0.143	0.032	1.491
Validation	Serotonin	biogenic amine	0.553	44.7%	−0.853	3.02 E-5	4.519
Validation	Arg	amino acid	0.864	13.6%	−0.210	3.09 E-3	2.510
Validation	PC aa C38:6	phosphatidylcholin (diacyl bond)	0.894	10.6%	−0.162	0.015	1.837
Validation	LPE a C22:6	lysophosphatidylethanolamines (acyl bond)	0.907	9.3%	−0.141	3.75 E-3	2.427
Validation	N-C26:1-Cer	ceramide	0.908	9.2%	−0.139	6.31 E-3	2.200
Validation	Tyr	amino acid	0.916	8.4%	−0.126	0.012	1.924
Validation	H1	hexoses	0.930	7.0%	−0.105	0.027	1.568
Validation	PE aa C36:0	phosphatidylethanolamines (diacyl bonds)	0.924	7.6%	−0.114	0.028	1.549
Validation	Lys	amino acid	0.938	6.2%	−0.093	0.029	1.537
Validation	Ile	amino acid	0.934	6.6%	−0.098	0.043	1.366
merged	Serotonin	biogenic amine	0.554	44.6%	−0.851	2.06 E-5	4.686
merged	Arg	amino acid	0.877	12.3%	−0.190	1.40 E-3	2.854
merged	PC aa C38:6	phosphatidylcholin (diacyl bond)	0.879	12.1%	−0.186	2.96 E-3	2.529
merged	PC aa C40:6	phosphatidylcholin (diacyl bond)	0.885	11.5%	−0.177	0.015	1.821
merged	PC aa C36:0	phosphatidylcholin (diacyl bond)	0.888	11.2%	−0.172	0.037	1.438
merged	H1	hexoses	0.902	9.8%	−0.149	1.16 E-4	3.936
merged	LPE a C22:6	lysophosphatidylethanolamines (acyl bond)	0.914	8.6%	−0.130	0.010	2.020
merged	PC aa C38:3	phosphatidylcholin (diacyl bond)	0.934	6.6%	−0.098	0.036	1.443
merged	Tyr	amino acid	0.937	6.3%	−0.095	0.027	1.566
merged	SM C24:0	sphingomyelin	0.945	5.5%	−0.082	0.041	1.388
merged	PC aa C36:4	phosphatidylcholin (diacyl bond)	0.948	5.2%	−0.077	0.033	1.482
merged	Ile	amino acid	0.949	5.1%	−0.075	0.048	1.323

Metabolites are ranked according to their ratio between genotypes CC and CT/TT with a cut-off for t-test p-values of 0.05. Results are given for the discovery study and the validation study, and also for a merged data set after data processing and exclusion of outliers.

The second study (validation study, n = 240) was analysed accordingly. The metabolomics assay confirmed serotonin as the top hit with the lowest metabolite ratio (0.55) and also with the lowest p-value (3.02 E-5) of all metabolites (Fig. 1), followed by the amino acid arginine and a PC (Table 2).

**Figure 2:**
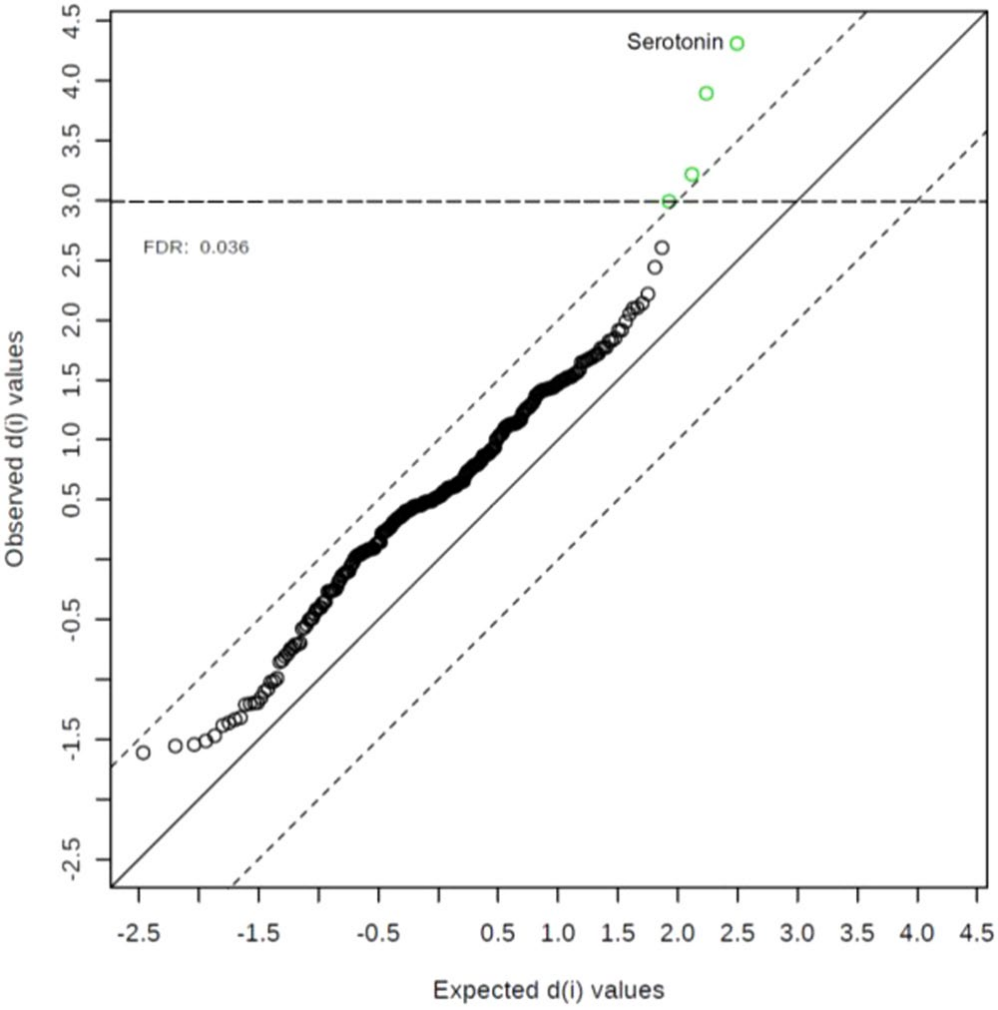
SAM plot. The plot depicts the observed relative differences versus the expected relative differences estimated by data permutation. The solid diagonal line indicates where these two measures are the same. The dotted lines are drawn at a distance of delta from the solid line. With a given delta of 1.0, four metabolites were identified to be significantly increased in the risk genotype (highlighted in green) according to an FDR of 3.59 E-2. Metabolites are listed in Supplementary Table 1.

After merging both studies (n = 394), the analysis of the merged population demonstrated again that serotonin is the metabolite with lowest ratio and lowest p-value (Table 2). Finally, the merged population was also analysed according to the Significance Analysis of Microarray (SAM) approach^17^ (Supplementary Table 1) providing estimates of the false discovery rate (FDR). Serotonin was verified as the top ranked metabolite out of four with significantly increased concentrations (Fig. 2**)**.

### Association between the rs7903146 risk variant and serotonin

**Figure 3:**
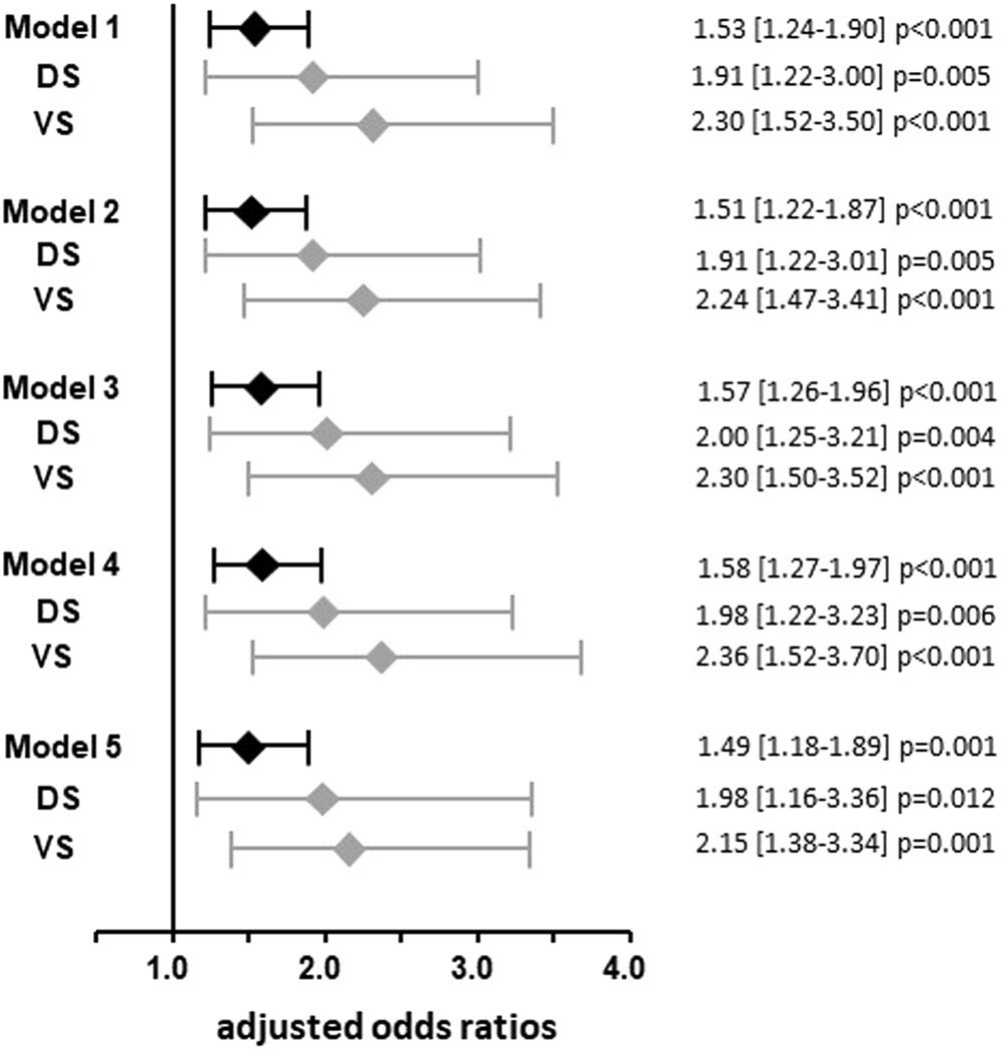
Association between rs7903146 genotype and serotonin. The forest plot depicts the adjusted odds ratios and 95% CI derived from binary logistic regression analysis for the association between TCF7L2 rs7903146 genotype and serum serotonin concentration as a continuous variable with stepwise adjustment. Adjustment model 1, a univariate model, represents the association between the genotype and serotonin. Model 2 represents the association between the genotype and serotonin with the type 2 diabetes status as covariate. Model 3 accounts for the parameters included in model 2 and, in addition, the covariates age, gender, and body mass index. Model 4 accounts for the parameters included in model 3 and, in addition, the hypertension status, the sig. CAD status, and the current smoking status. Model 5 accounts for the parameters included in model 4 and, in addition, arginine and the phosphatidylcholine PC aa C38:6. DS denotes discovery study, VS validation study.

Serotonin concentration was significantly higher in subjects with the risk allele (CT/TT) than in those carrying no risk allele (CC). In addition, it increased according to CC-CT-TT genotypes in patients without type 2 diabetes, but not in subjects with type 2 diabetes (Supplementary Fig. 2). Logistic regression analyses revealed that serotonin was significantly associated with the risk allele of rs7903146 even after adjustment for type 2 diabetes in the discovery study (ad. OR = 1.91 [95% CI 1.22–3.01], p = 0.005) and the validation study (ad. OR = 2.24 [95% CI 1.47–3.41], p < 0.001) as well. The factor “type 2 diabetes by rs7903146” was not significantly associated with serotonin in neither of both studies (p = 0.910 and p = 0.830) in analysis of covariance, indicating that there is no significant impact of the diabetic state on the association between rs7903146 and serotonin. In further multivariate regression analyses including adjustment for covariates age, gender, body mass index (BMI), hypertension, significant coronary artery disease (sig. CAD), smoking status, and even top2 and top3 identified metabolites (arginine and PC aa C38.6, Table 2), serotonin remained significantly associated with rs7903146 (Fig. 3) in the discovery study (adj. OR = 1.98 [95% CI 1.16–3.36], p = 0.012) and in the validation study (adj. OR = 2.15 [95% CI 1.38–3.34], p = 0.001). Having pooled patients of both studies, a similar result was seen in the merged study population (adj. OR = 1.49 [95% CI 1.18–1.89], p = 0.001). In addition, even if patients with type 2 diabetes (n = 125) were excluded from analysis, the risk allele of rs7903146 remained significantly associated with serotonin in the fully adjusted model (adj. OR = 1.51 [95% CI 1.14–2.01], p = 0.005) and this was also true in the discovery (adj. OR = 2.15 [95% CI 1.16–3.97], p = 0.015) and validation study (adj. OR = 2.07 [95% CI 1.23–3.51], p = 0.007); Supplementary Fig. 3). Furthermore, not even the exclusion of patients being treated with serotonin-impacting drugs (n = 44, Supplementary Table 2) did abrogate the significant association between the risk genotype and serotonin (pooled adj. OR = 1.34 [95% CI 1.03–1.73], p = 0.027; discovery study adj. OR = 1.80 [95% CI 1.00–3.22], p = 0.049; validation study adj. OR = 1.92 [95% CI 1.19–3.10], p = 0.008; Supplementary Fig. 4).

**Figure 4:**
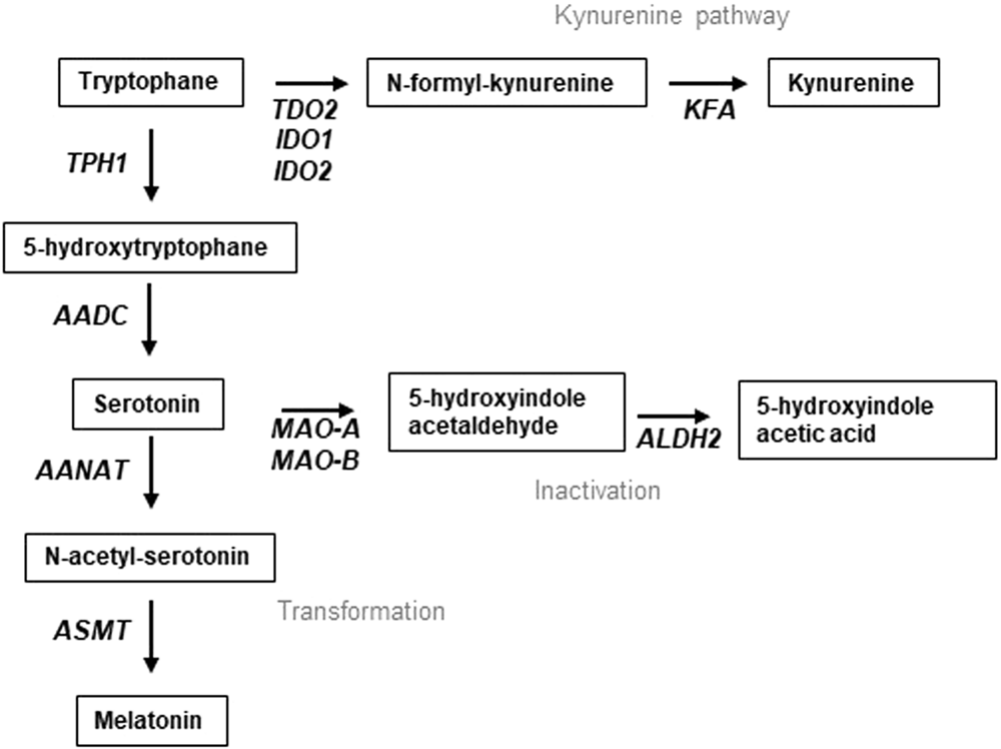
Main metabolic pathways in serotonin biosynthesis. AADC denotes aromatic L-amino-acid decarboxylase, AANAT aralkylamine N-acetyltransferase, ALDH aldehyde dehydrogenase, ASMT N-acetylserotonin O-methyltransferase, IDO indoleamine-pyrrole 2,3 dioxygenase, KFA Kynurenine formamidase, MAO Monoaminooxidase, TDO tryptophan 2,3 dioxygenase, TPH represents tryptophane hydroxylase.

With respect to the tryptophan (Trp) and serotonin metabolism (Fig. 4), we also compared the Trp to serotonin and the Trp to kynurenine ratio. We found a significant association between the Trp-serotonin ratio and rs7903146 but not between the Trp-kynurenine ratio and rs7903146 in the discovery study and the validation study, and also in the merged population (Supplementary Fig. 5).

### Validation of association between serotonin and type 2 diabetes

We went on to verify whether serotonin concentrations were also elevated in diabetic subjects of our study population as reported previously by others^1,2^. We could confirm that serotonin concentration as determined by mass spectroscopy was significantly higher in diabetic patients (n = 125) than in non-diabetic patients of our merged population (n = 269; 0.112 vs. 0.074 µmol/l, p = 0.013). In addition, we also compared the Trp to serotonin, the Trp to kynurenine ratio, and the kynurenine concentration in diabetic and non-diabetic patients. Coherently, the Trp-serotonin ratio was significantly lower in diabetic than in non-diabetic patients (1.70 E + 3 vs. 2.21 E + 3, p = 0.011), whereas no significant difference was seen with respect to the Trp-kynurenine ratio (14.9 vs. 15.2, p = 0.306) or the kynurenine concentration only (4.5 vs. 4.4 µmol/l, p = 0.466).

### Validation of association between the rs7903146 risk variant and type 2 diabetes

Analysing all 1660 genotyped patients in the total study, we found that risk allele carriers (n = 872) had a significantly higher fasting glucose (115.8 vs.109.3 mg/dl, p = 0.007), post-challenge glucose (147.1 vs.135.2 mg/dl, p = 0.036), and HbA1c (6.2% (44 mmol/mol) vs. 6.0% (42 mmol/mol), p < 0.001), than patients who were (C/C) homozygous (n = 788). In accordance with previous data^3,6–9^, the prevalence of type 2 diabetes was significantly higher in CT/TT-carriers than in CC-carriers (30.8 vs. 25.8%, p = 0.022). However, fasting insulin was not significantly different between CT/TT- and CC-carriers (12.6 vs. 12.3 µU/ml, p = 0.229). Furthermore, there was no significant difference with respect to BMI (27.4 vs. 27.7, p = 0.358), triglycerides (145 mg/dl (1.6 mmol/L) vs. 154 mg/dl (1.7 mmol/L), p = 0.099), LDL-C (128 mg/dl (3.30 mmol/L) vs. 130 mg/dl (3.36 mmol/L), p = 0.199), or HDL-C (54 mg/dl (1.39 mmol/L) vs. 53 mg/dl (1.37 mmol/L), p = 0.273).

### Genotyping of the TCF7L2 gene locus

As genome wide association studies (GWAS) with serotonin as phenotype did not identify a genomic region with genome-wide significance, we focused on the locus of the TCF7L2 gene (position 112.910 kb and 113.278 kb (GRCh38.7); chromosome 10) and obtained a hint for an association between the TCF7L2 gene and serotonin concentration (Supplementary Fig. 6). We thus analysed the TCF7L2 gene locus which comprises 286 SNPs on the Cardio-Metabo chip (Illumina). After Pruning, 130 SNPs remained and were used in tagging analysis (Supplementary Fig. 7). Tagging analysis with a threshold of r^2^ < 0.9 for linkage disequilibrium (LD) revealed 19 unlinked SNPs, which cover the mentioned genomic region. These SNPs were analysed, and we found that 8 of these 19 SNPs were significantly associated with serotonin after FDR correction for multiple testing (Supplementary Table 3).

### Promotor analysis of genes involved in serotonin metabolism

In order to analyse promotors of genes involved in serotonin biosynthesis and metabolism (Fig. 4), we applied in silico binding site prediction for transcription factor TCF7L2 using the Match search algorithm^18^ together with the TRANSFAC database. Proximal promoter sequences of tryptophan hydroxylase 1 (TPH1), tryptophan 2,3-dioxygenase 2 (TDO2), indoleamine-pyrrole 2,3-dioxygenase 1 and 2 (IDO1, IDO2), aromatic L-amino acid decarboxylase (AADC), monoamine oxidases A and B (MAO-A, MAO-B), aldehyde dehydrogenase 2 (ALDH2), aralkylamine *N*-acetyltransferase (AANAT), N-acetylserotonin O-methyltransferase (ASMT), were analysed. By doing so we applied a user-defined profile of high quality matrices (Supplementary Table 4). We could identify several TCF7L2-specific binding sites in every promoter (Supplementary Fig. 8) including the TCF7L2 promoter, for which binding is proven by chromatin immunoprecipitation^19^.

## Discussion

### TCF7L2 polymorphism rs7903146 is associated with serotonin and glycerolphospholipid concentration in targeted metabolomics

In the present study, we have applied targeted metabolomics on 154 serum samples in a discovery study and replicated this analysis 14 months later using 240 samples in a validation study. In both studies, we found that circulating serotonin was the metabolite with the highest values in individuals with the type 2 diabetes risk variant rs7903146 in TCF7L2 compared to patients without the variant. We also found that further common variants within the genomic region of TCF7L2 gene, which are not genetically linked with rs7903146, were significantly associated with serum serotonin concentrations. In addition, serotonin concentration was significantly associated with the prevalence of type 2 diabetes. Of note, the association between serotonin and rs7903146 was independent from patients’ type 2 diabetes status and was also found in patients without type 2 diabetes.

Whereas a previous non-targeted metabolomics assay did not reveal any metabolic difference between rs7903146 genotypes in 30 participants^16^, targeted metabolomics, given its methodical differences^20^, could demonstrate alterations of phospholipid metabolism in response to challenge tests in subjects carrying the TCF7L2 risk allele (n = 17) compared to those without the risk allele (n = 24)^21^. These results have been suggested to reflect a genotype-mediated link to early metabolic abnormalities that precede the development of disturbed glucose tolerance^21^. In our study, comprising nearly 400 participants, we have identified -besides serotonin - several glycerolphospholipids to be significantly associated with rs7903146 as well, thus corroborating these previous data.

### TCF7L2 is part of the Wnt signalling and polymorphisms in TCF7L2 are associated with type 2 diabetes

TCF7L2 is a major transcription factor of the canonical Wnt signalling pathway. The key effector of the Wnt signalling pathway is the bipartite transcription factor β-catenin/TCF. It is formed by free β-catenin and a member of the TCF protein family, which includes TCF7L2^22^. The prerequisite for Wnt signalling is the binding of a Wnt ligand to the Frizzled receptor and the co-receptor LDL receptor-related protein 5 (LRP5) or LRP6, preventing degradation of β-catenin and allowing formation of the β-catenin/TCF complex and activation of downstream target genes^22^.

Wnt signalling is involved in regulating numerous processes including metabolic homeostasis in general, bone formation^23^, hormone gene expression and pancreatic β-cell proliferation^24^. Particularly, TCF7L2 is known as a major type 2 diabetes susceptibility gene^3,7^, which is also linked to diabetic coronary atherosclerosis^9^. A recent GWAS comprising 62,892 type 2 diabetes cases and 596,424 controls of European ancestry, identified polymorphism rs7903146 as the variant with the highest association, out of 16 million genetic variants^25^. Thus the overall mechanism, how transcription factor TCF7L2 and in particular rs7903146 impacts the metabolic state and is associated with diabetes has been intensively discussed in the past. Although there are some hypotheses based on incretin, glucose disposal, and insulin signalling data^4,22,26,27^, the connecting mechanism between diabetes and TCF7L2 still remained far from being thoroughly understood^22^.

More recently, the rs7903146 variant in the TCF7L2 gene has been reported to increase the risk of prediabetes and type 2 diabetes in obese subjects by declining insulin sensitivity and β-cell function, and also predicts the development of prediabetes and diabetes over time^28^. Reviewing 153 meta-analyses, Amare *et al*. found a genetic overlap between cardiometabolic diseases and mood disorders in 24 genes, including TCF7L2^29^. Pathway analysis identified serotonin receptor signalling consistently being within the top ranked hits^29^. Thus the identification of elevated serotonin in rs7903146 CT/TT allele carriers in our study offers a new way to explain the link between TCF7L2 and diabetes.

### Serotonin is elevated in diabetes

Serotonin is synthesized from tryptophan, the least abundant essential amino acid in mammals. It is regulated by the rate limiting enzyme tryptophan hydroxylase (TPH). THP is not only found in the brain (TPH2) but also in enterochromaffin cells in the intestine (TPH1)^30^. Biosynthesis of serotonin is thus dependent on dietary tryptophane intake and also on the kynurenine pathway as there is a balance between the kynurenine and serotonin pathways^31^ (Fig. 4). Similarly, kynurenine concentration is influenced by tryptophan intake as well. Therefore, the kynurenine to tryptophan ratio is better suited to represent tryptophan catabolism than the absolute concentration of kynurenine^32^. Serotonin, on the other hand, can be transformed to melatonin or catabolised to inactive molecules found in urine. It is a physiological modulator and thus impacts of several processes including mood and sleep, regulation of appetite, body temperature, and metabolism^29^. Lorcaserin, a brain-specific serotonin-agonist^33^ has recently demonstrated its anorectic potential promoting weight loss with no excess in cardiovascular events but a reduced rate of new onset or progressive renal impairment in the CAMELLIA-TIMI 61 Trial^34,35^. However, serotonin not only acts in the brain. Ninety-five percent of serotonin is produced in the periphery^2^, in particular in the gut^36^ and its synthesis is modulated by gut microbiota^37^. Of note, serotonin does not pass the blood-brain barrier and the two major pools, brain and periphery, remain separated^2^. In the periphery, it promotes inflammation^38^, inhibits activity of brown adipose tissue, and is elevated in obesity^39^. Moreover, it regulates glucose homeostasis, hepatic gluconeogenesis, and mobilization of hepatic free fatty acids^40^, and thus has direct implication for metabolic disorders such as diabetes^41^. Apart from the intestine, serotonin is also synthesized in pancreatic β-cells^42^, stored along with insulin-granules, and is released upon glucose stimulation^43^. Serotonin is also a strong paracrine regulator of α-cell activity^44^. However, the mechanisms that drive altered serotonin signalling in the pancreatic cells remain unclear^41^.

Nevertheless, with respect to its concentration in the circulation, serotonin is known to be increased in individuals with diabetes^41^. In accordance, our study found significantly higher serotonin concentrations in diabetic patients compared to patients without diabetes. Moreover, there is a well-known increased risk of diabetes in patients suffering from depression and taking e.g. serotonin reuptake inhibitors increasing the extracellular level of serotonin and targeting serotonin signalling^45^. However, we could rule out the confounding of our results by serotonin-impacting medication in a subgroup analysis.

### TCF7L2 rs7903146 polymorphism is associated with serotonin, independent from diabetes, but it is not associated with kynurenine

We found that the association between rs7903146 and serotonin is independent of and, therefrom not confounded by the diabetic status of the patients. This association was found both in the discovery study and the validation study. In addition, we found that 8 of 19 tagging SNPs covering the TCF7L2 locus were significantly associated with serotonin, even after correction for multiple testing.

We also demonstrated that this association was specific for serotonin and did not apply to the alternative tryptophan metabolite kynurenine, precluding upstream effects e.g. tryptophan intake. Thus, the higher serotonin concentration probably results from decreased processing of serotonin.

In that context, we also found several binding sites for TCF7L2 in genes involved in serotonin biosynthesis. However, it has to be mentioned that validity of in silico only data is quite limited; thus, this is no proof for a direct binding of the transcription factor TCF7L2 to respective promoter sequences, but at the same time it precludes that direct binding is prevented. As there are no experimental data available, apart from binding of TCF7L2 to its own promoter region^19^, these genes may be candidates for functional studies in the future.

### Wnt signalling impacts serotonin concentration and thus may be a missing link between polymorphisms in TCF7L2 and diabetes

The present study’s data have clearly demonstrated that there is a significant association between the major transcription factor of the Wnt signalling pathway, TCF7L2, and serotonin concentration. Thus our findings are, in part, corroborated by previous data in the context of bone turnover, suggesting as well that Wnt signalling is most likely involved in serotonin expression^46^. This is also in line with more recent findings in enteroendocrine cell lineages, in which serotonin biosynthesis has been demonstrated to be regulated by LMX1A^47^. LMX1A, on the other hand, is regulated by Wnt signalling and builds an autoregulatory loop with Wnt1^48,49^ during embryogenesis. Taken together all these data support the hypothesis that serotonin might be involved, at least in part, in the impact of Wnt signalling on metabolic homeostasis and type 2 diabetes^22^. These data provide a possible link between polymorphisms in TCF7L2 and serotonin and suggest genes involved in serotonin metabolism as candidate genes for functional studies in the future.

### Limitations and open questions

The current study has its limitations. It has to be mentioned that there are further mechanism by which TCF7L2 might impact the diabetes risk, and some of these mechanism may be independent from Wnt signalling^22^. Apart from that, there are also other mechanisms probably contributing to and being involved in the regulation of the serotonin level and development of diabetes e.g. the gut microbiome^41^ or mutation of the tryptophan hydroxylase gene^43^. Moreover, an isotope labelling cell experiment would be well-suited for confirming that serotonin is from tryptophane in cells with rs7903146 genotype and that serotonin production in cells with rs7903146 is different from that seen in wt cells. Second, our study patients are a selected group of coronary patients and thus, do not necessarily reflect the general population. However, this study population, which is at a very high risk of cardiovascular complications, is clinically very important.

## Conclusion

This study is currently the largest investigation on the impact of rs7903146 on the metabolome. It provides a first hint that the well-known impact of the TCF7L2 polymorphism rs7903146 on diabetes risk may, at least in part, be dependent on serotonin regulation mediated by the Wnt signalling pathway. Thus these findings may help to shed light on the mechanism that increases circulating serotonin in the context of metabolic dysfunction and type 2 diabetes, which has been obscure until now. Finally, the presented link between TCF7L2 and serotonin is biologically plausible, and the association between TCF7L2 and diabetes on the one hand and the association between serotonin and diabetes on the other hand are already known. Our results should foster more interest into the described hypothesis.

## Methods

### Study subjects and metabolomic analysis

For the discovery phase study in December 2009, we randomly and consecutively selected and analysed 154 patient samples from a total study population of 1660 angiographied and genotyped patients of Caucasian origin which have been recruited between 1999 and 2009. Further details are given in the supplementary part or were reported previously^9^. For the validation study, 240 samples were consecutively enrolled from the same total study population and analysed in February 2011.

For both the metabolomics assays, we applied a targeted quantitative metabolomic approach to analyse the serum samples (BIOCRATES Life Sciences AG, Austria) using liquid chromatography (LC), - mass spectroscopy (MS), and flow injection analysis (FIA) - MS respectively. In total, 539 compounds were analysed in 394 serum samples. For data pre-processing, metabolites with missing values of at least 20% were removed. Remaining missing values were imputed by half of the minimum positive value in the original data set (program default setting). After that, non-informative variables of very small values, close to baseline or detection limit, were detected according to median and filtered. Then, data were normalised by the reference feature BMI due to its association with rs7903146^4^, followed by a transformation (log2) to correct for heteroscedasticity, and finally by Pareto-scaling to minimize the effect of small noisy variables. Outlier detection was performed using principal component analysis (PCA, Supplementary Figure 1) and respective samples (n = 5 in the discovery study; n = 1 in the validation study) were excluded from further metabolomic analysis. Multiple testing was addressed by calculating q-values^50^ and using significance analysis of microarrays (SAM)^17^. Data were finally analysed using MetaboAnalyst software version 3.0 (http://www.metaboanalyst.ca/).

### Basic laboratory tests, genotyping, and transcription factor analysis

A detailed description of laboratory, genotyping and transcription factor analyses is given in the supplemental methods section. In short, venous blood samples were collected from fasted subjects and basic laboratory measurements were immediately performed. DNA was extracted from all 1660 patient samples and SNPs in cardiovascular and metabolism genes were characterised using the Illumina Cardio-Metabo chip technology (Illumina Inc., San Diego, CA)^51^. Transcription factor analysis was done in silico applying the Match search algorithm^18^ in combination with the professional version of TRANSFAC database (release 2018.2), implemented in the geneXplain platform (http://genexplain.com/genexplain-platform/; GeneXplain, Wolfenbüttel, Germany).

### Statistics

Differences in baseline characteristics were tested for statistical significance with the Chi-squared tests for categorical and Jonckheere-Terpstra tests for continuous variables, respectively. Correlation analyses were performed calculating non-parametric Spearman rank correlation coefficients. For comparing continuous or categorical variables we used Wilcoxon and McNemar tests, respectively. T-test was used only in terms of metabolomic analysis of normalised and logarithmised (log2) data. For logistic regression, data were z-transformed and data coming from LC-MS and FIA-MS were logarithmised (log2) before transformation, respectively. All results are given as mean ± standard deviation (SD); p-values < 0.05 were considered significant, if not otherwise specified (GWAs). All data were analysed according to complete-case analysis.

All statistical analyses were performed with SPSS 21.0, SPSS Sample Power 3.0 (SPSS, Inc., Chicago, IL), PLINK version 1.07; (http://zzz.bwh.harvard.edu/plink/download.shtml), and R statistical software version 3.2.3 (http://www.r-project.org).

The present study was performed in accordance with the Declaration of Helsinki. The study, all including research, and all methods adhere to relevant guidelines and regulations and have been approved by the Ethics Committee of the University of Innsbruck. Written informed consent was given by all participants.

### Prior presentation

Data have been presented during the 78^th^ ADA scientific sessions in Orlando.

## Supplementary information

Supplemental dataset

## Data Availability

The datasets generated during and/or analysed during the current study are available from the corresponding author on reasonable request.

## References

[1] Steinberg GR (2018). Cellular Energy Sensing and Metabolism-Implications for Treating Diabetes: The 2017 Outstanding Scientific Achievement Award Lecture. Diabetes.

[2] El-Merahbi R, Löffler M, Mayer A, Sumara G (2015). The roles of peripheral serotonin in metabolic homeostasis. FEBS Lett..

[3] Saxena R (2006). Common Single Nucleotide Polymorphisms in TCF7L2 Are Reproducibly Associated With Type 2 Diabetes and Reduce the Insulin Response to Glucose in Nondiabetic Individuals. Diabetes.

[4] Lyssenko V (2007). Mechanisms by which common variants in the TCF7L2 gene increase risk of type 2 diabetes. J. Clin. Invest..

[5] Cauchi S (2007). TCF7L2 is reproducibly associated with type 2 diabetes in various ethnic groups: a global meta-analysis. J. Mol. Med..

[6] Grant SFA (2006). Variant of transcription factor 7-like 2 (TCF7L2) gene confers risk of type 2 diabetes. Nat. Genet..

[7] Florez JC (2006). TCF7L2 Polymorphisms and Progression to Diabetes in the Diabetes Prevention Program. N. Engl. J. Med..

[8] Loos RJF (2007). TCF7L2 Polymorphisms Modulate Proinsulin Levels and -Cell Function in a British Europid Population. Diabetes.

[9] Muendlein A (2011). Single nucleotide polymorphisms of TCF7L2 are linked to diabetic coronary atherosclerosis. PLoS One.

[10] Duval A, Busson-Leconiat M, Berger R, Hamelin R (2000). Assignment of the TCF-4 gene (TCF7L2) to human chromosome band 10q25.3. Cytogenet. Genome Res..

[11] Mulholland DJ, Dedhar S, Coetzee GA, Nelson CC (2005). Interaction of Nuclear Receptors with the Wnt/β-Catenin/Tcf Signaling Axis: Wnt You Like to Know?. Endocr. Rev..

[12] da Silva Xavier G (2012). Abnormal glucose tolerance and insulin secretion in pancreas-specific Tcf7l2-null mice. Diabetologia.

[13] Pearson ER (2009). Translating TCF7L2: from gene to function. Diabetologia.

[14] Florez JC (2008). Newly identified loci highlight beta cell dysfunction as a key cause of type 2 diabetes: Where are the insulin resistance genes?. Diabetologia.

[15] Shah SH, Newgard CB (2015). Integrated Metabolomics and Genomics. Circ. Cardiovasc. Genet..

[16] Wagner R (2015). Clinical and non-targeted metabolomic profiling of homozygous carriers of Transcription Factor 7-like 2 variant rs7903146. Sci. Rep..

[17] Tusher VG, Tibshirani R, Chu G (2001). Significance analysis of microarrays applied to the ionizing radiation response. Proc. Natl. Acad. Sci..

[18] Kel AE (2003). MATCH: A tool for searching transcription factor binding sites in DNA sequences. Nucleic Acids Res..

[19] Verzi MP (2010). TCF4 and CDX2, major transcription factors for intestinal function, converge on the same cis-regulatory regions. Proc. Natl. Acad. Sci..

[20] Roberts Lee D., Souza Amanda L., Gerszten Robert E., Clish Clary B. (2012). Targeted Metabolomics. Current Protocols in Molecular Biology.

[21] Then C (2013). Plasma Metabolomics Reveal Alterations of Sphingo- and Glycerophospholipid Levels in Non-Diabetic Carriers of the Transcription Factor 7-Like 2 Polymorphism rs7903146. PLoS One.

[22] Ip W, Chiang Y, Jin T (2012). The involvement of the wnt signaling pathway and TCF7L2 in diabetes mellitus: The current understanding, dispute, and perspective. Cell Biosci..

[23] Rachner TD, Khosla S, Hofbauer LC (2011). Osteoporosis: now and the future. Lancet.

[24] Pujadas G (2016). Wnt9a deficiency discloses a repressive role of Tcf7l2 on endocrine differentiation in the embryonic pancreas. Sci. Rep..

[25] Xue A (2018). Genome-wide association analyses identify 143 risk variants and putative regulatory mechanisms for type 2 diabetes. Nat. Commun..

[26] Villareal DT (2010). TCF7L2 Variant rs7903146 Affects the Risk of Type 2 Diabetes by Modulating Incretin Action. Diabetes.

[27] Chang Y-C (2010). TCF7L2 genetic variants and progression to diabetes in the Chinese population: pleiotropic effects on insulin secretion and insulin resistance. J. Mol. Med..

[28] Cropano C (2017). The rs7903146 Variant in the *TCF7L2* Gene Increases the Risk of Prediabetes/Type 2 Diabetes in Obese Adolescents by Impairing β-Cell Function and Hepatic Insulin Sensitivity. Diabetes Care.

[29] Amare AT, Schubert KO, Klingler-Hoffmann M, Cohen-Woods S, Baune BT (2017). The genetic overlap between mood disorders and cardiometabolic diseases: a systematic review of genome wide and candidate gene studies. Transl. Psychiatry.

[30] Walther DJ (2003). Synthesis of Serotonin by a Second Tryptophan Hydroxylase Isoform. Science..

[31] Li Y (2017). Regulating the balance between the kynurenine and serotonin pathways of tryptophan metabolism. FEBS J.

[32] Rebnord EW (2017). The kynurenine:tryptophan ratio as a predictor of incident type 2 diabetes mellitus in individuals with coronary artery disease. Diabetologia.

[33] Meltzer HY, Roth BL (2013). Lorcaserin and pimavanserin: emerging selectivity of serotonin receptor subtype–targeted drugs. J. Clin. Invest..

[34] Scirica BM (2019). Lorcaserin and Renal Outcomes in Obese and Overweight Patients in the CAMELLIA-TIMI 61 Trial. Circulation.

[35] Bohula EA (2018). Cardiovascular Safety of Lorcaserin in Overweight or Obese Patients. N. Engl. J. Med..

[36] Gershon MD (2013). 5-Hydroxytryptamine (serotonin) in the gastrointestinal tract. Curr. Opin. Endocrinol. Diabetes Obes..

[37] Yano JM (2015). Indigenous bacteria from the gut microbiota regulate host serotonin biosynthesis. Cell.

[38] Ghia J (2009). Serotonin Has a Key Role in Pathogenesis of Experimental Colitis. Gastroenterology.

[39] Crane JD (2015). Inhibiting peripheral serotonin synthesis reduces obesity and metabolic dysfunction by promoting brown adipose tissue thermogenesis. Nat. Med..

[40] Sumara G, Sumara O, Kim JK, Karsenty G (2012). Gut-Derived Serotonin Is a Multifunctional Determinant to Fasting Adaptation. Cell Metab..

[41] Martin AM (2017). The Diverse Metabolic Roles of Peripheral Serotonin. Endocrinology.

[42] Schraenen A (2010). Placental lactogens induce serotonin biosynthesis in a subset of mouse beta cells during pregnancy. Diabetologia.

[43] Paulmann N (2009). Intracellular Serotonin Modulates Insulin Secretion from Pancreatic β-Cells by Protein Serotonylation. PLoS Biol..

[44] Almaça J (2016). Human Beta Cells Produce and Release Serotonin to Inhibit Glucagon Secretion from Alpha Cells. Cell Rep..

[45] Brown LC, Majumdar SR, Johnson JA (2008). Type of antidepressant therapy and risk of type 2 diabetes in people with depression. Diabetes Res. Clin. Pract..

[46] Galli C, Macaluso G, Passeri G (2013). Serotonin: a novel bone mass controller may have implications for alveolar bone. J. Negat. Results Biomed..

[47] Gross S (2016). The novel enterochromaffin marker Lmx1a regulates serotonin biosynthesis in enteroendocrine cell lineages downstream of Nkx2.2. Development.

[48] Anderegg A (2013). An Lmx1b-miR135a2 Regulatory Circuit Modulates Wnt1/Wnt Signaling and Determines the Size of the Midbrain Dopaminergic Progenitor Pool. PLoS Genet..

[49] Chung S (2009). Wnt1-lmx1a forms a novel autoregulatory loop and controls midbrain dopaminergic differentiation synergistically with the SHH-FoxA2 pathway. Cell Stem Cell.

[50] Storey JD (2002). A direct approach to false discovery rates. J. R. Stat. Soc. Ser. B (Statistical Methodol..

[51] Voight BF (2012). The Metabochip, a Custom Genotyping Array for Genetic Studies of Metabolic, Cardiovascular, and Anthropometric Traits. PLoS Genet..

