# Supplementary material for: Serotonin is elevated in risk-genotype carriers of TCF7L2 - rs7903146
Source: Sci Rep. 2019 Sep 6;9:12863. doi: 10.1038/s41598-019-49347-y (PMC6731216; doi:10.1038/s41598-019-49347-y)
Supplement: Supplementary file 1 — Supplemental dataset [file 41598_2019_49347_MOESM1_ESM.pdf]

Supplemental data

Serotonin is elevated in risk-genotype carriers of TCF7L2 - rs7903146

Andreas LEIHERER PhD [1,3,4], Axel MUENDLEIN PhD [1,3], Christoph H. SAELY MD [1,2,3], Peter FRAUNBERGER MD [3,4], and Heinz DREXEL MD [1,3,5,6*]

[1] *Vorarlberg Institute for Vascular Investigation and Treatment (VIVIT), Feldkirch, Austria*
[2] *Department of Medicine and Cardiology, Academic Teaching Hospital Feldkirch, Feldkirch, Austria*
[3] *Private University of the Principality of Liechtenstein, Triesen, Liechtenstein*
[4] *Medical Central Laboratories, Feldkirch, Austria*
[5] *Drexel University College of Medicine, Philadelphia, PA, USA*
[6] *Division of Angiology, Swiss Cardiovascular Center, University Hospital of Bern, Switzerland*

[*] *Corresponding author at: Vorarlberg Institute for Vascular Investigation and Treatment (VIVIT), Academic Teaching Hospital Feldkirch, Carinagasse 47, A-6807 Feldkirch, Austria.*
tel.: +43 5522 303 6902;
fax: +43 5522 303 7533;.
email address: labor@vivit.at

A) Supplemental tables

Metabolite	class	d-value	SD	p-value	q-value
Serotonin*	biogenic amine	4.312	0.117	<1.00E-04	<1.00E-04
H1*	hexoses	3.895	0.066	1.08E-04	0.011
Arg*	amino acid	3.218	0.077	1.30E-03	0.080
PC aa C38:6*	phosphatidylcholine (diacyl bond)	2.991	0.078	2.67E-03	0.107
LPE a C22:6	lysophosphatidylethanolamine (acyl bond)	2.605	0.072	0.009	0.318
PC aa C40:6	phosphatidylcholine (diacyl bond)	2.441	0.081	0.014	0.418
Tyr	amino acid	2.218	0.065	0.026	0.595
PC aa C36:4	phosphatidylcholine (diacyl bond)	2.140	0.073	0.032	0.595
PC aa C38:3	phosphatidylcholine (diacyl bond)	2.104	0.134	0.035	0.595
PC aa C36:0	phosphatidylcholine (diacyl bond)	2.098	0.127	0.035	0.595

Supplementary table 1: Top ten metabolites associated with the TCF7L2 rs7903146 genotype according to SAM-test. Metabolites were identified according to the Significance Analysis of Microarray (SAM) approach. The top ten metabolites are ranked according to their d-value (T-statistic-value), p-value, and q-value (FDR-adjusted p-value) respectively. Results are given for the merged data set of the discovery and validation study after data processing and exclusion of outliers. Four metabolites have been identified to be significantly positive (asterisks) with an FDR of 3.59 E-2 and a delta of 1.0 (depicted in **figure 2**).

Treatment regimens				Trade names			Number of patients
Amitriptylin (10mg)	Bromazepam (3mg)			Saroten	Bromazepam		1
Amitriptylin (10mg)				Saroten			1
Amitriptylin (25mg)				Saroten			1
Citalopram (20mg)	Lorazepam (1mg)			Seropram	Temesta		1
Citalopram (20mg)	Melitracen (10mg)	Flupentixol (0.5mg)	Mirtazapin (30mg)	Seropram	Deanxit	Mirtabene	1
Citalopram (20mg)	Protipendyl (80mg)			Seropram	Dominal		1
Citalopram (20mg)	Trazodon (75mg)			Citalopram	Trittico		1
Citalopram (20mg)	Zolpidem (10mg)			Cipralex	Ivadal		1
Citalopram (10mg)				Cipralex			4
Citalopram (20mg)				Seropram, Pram			10
Fluoxetin (20mg)	Trazodon (75mg)			Fluoxetin	Trittico		1
Fluoxetin (20mg)				Fluctine			1
Fluoxetin (10mg)				Fluoxetin			1
Melitracen (10mg)	Flupentixol (0.5mg)			Deanxit			5
Paroxetin (20mg)	Trazodon (150mg)			Seroxat	Trittico		1
Paroxetin (20mg)				Seroxat			4
Sertralin (50mg)	Alprazolam (1mg)			Gladem	Xanor		1
Trazodon (150mg)	Citalopram (40mg)			Trittico	Pram		1
Trazodon (75mg)				Trittico			1
Trazodon (150mg)				Trittico			6
							total 44

Supplementary table 2: List of medication regimens which were prescribed to study participants with mood disorders (n=44). The table presents all medications which are considered to alter serotonin-concentration and which have been prescribed and consumed to treat mood disorders in 44 patients.

	SNP	position	β-value	p-value	BH FDR
1	**rs55972445**	113023031	0.124	9.21E-04	1.45E-03
2	**rs61872786**	113046938	0.121	1.75E-03	1.45E-03
3	**rs7903146**	112998590	0.103	2.29E-03	1.45E-03
4	**rs10128255**	112983076	-0.088	3.91E-03	1.86E-03
5	**rs7901695**	112994329	0.094	5.15E-03	1.96E-03
6	**rs12255372**	113049143	0.091	8.34E-03	2.64E-03
7	**rs4077527**	113053282	0.080	1.32E-03	3.53E-03
8	**rs187374308**	112987883	0.080	2.09E-03	4.99E-03
9	rs4073980	112986821	0.070	3.04E-03	6.33E-03
10	rs35519679	113058995	0.070	5.27E-03	0.107
11	rs7085989	113062980	0.054	8.59E-03	0.149
12	rs290483	113155455	-0.051	0.121	0.192
13	rs11196175	112976855	0.051	0.135	0.197
14	rs35936842	113059013	0.043	0.178	0.242
15	rs7085532	113099704	0.032	0.338	0.428
16	rs4918796	113120583	0.019	0.600	0.713
17	rs7917983	112973123	-0.010	0.742	0.829
18	rs4639863	113094624	0.006	0.873	0.9225
19	rs10885414	113101545	0.002	0.953	0.953

Supplementary table 3: Association of 19 tagging SNPs for TCF7L2 genomic region with serotonin concentration. The table presents tagging SNPs for TCF7L2 genomic region and their association with serotonin concentration. The position refers to chromosome 10 according to GRCh38.7. SNPs are ranked according to their p-value with the respective adjusted p-values according to Benjamini&Hochberg for FDR correction for multiple comparisons and β-values. SNPs being significantly associated according to adjusted p-values are highlighted in bold type.

Gene (Entrez ID)	Matrix name	Factor name	Position	Core score	Matrix score	Matched sequence
TPH1	V$TCF4_04	TCF4	18041120 (+)	1.000	0.958	ccCACCTtgc
(7166)	V$TCF4_04	TCF4	18041197 (+)	1.000	0.957	ctCACCTtct
	V$TCF4_Q5_01	TCF-4	18041359 (-)	1.000	0.973	ctaCTTTGgt
	V$TCF4_Q5_02	TCF-4	18041361 (-)	1.000	0.954	aCTTTGgtat
	V$TCF4_04	TCF4	18041378 (+)	1.000	0.953	acCACCTatt
TDO2	V$TCF7L2_07	TCF-4	155854466 (-)	1.000	0.886	agtcTTTGAcccat
(6999)	V$TCF4_Q5_02	TCF-4	155854468 (-)	1.000	0.975	tCTTTGaccc
	V$TCF7L2_06	TCF-4	155854468 (+)	1.000	0.934	tCTTTGacc
	V$TCF4_04	TCF4	155854500 (+)	0.873	0.876	tgCACATgta
	V$TCF4_04	TCF4	155854551 (+)	1.000	0.954	gaCACCTaat
	V$TCF4_06	TCF-4	155854575 (-)	0.915	0.884	caatTTAAAggaa
	V$TCF4_04	TCF4	155854728 (-)	0.878	0.882	tgcAGGAGct
IDO1	V$TCF4_Q5_02	TCF-4	39901886 (+)	1.000	0.955	ctacCAAAGa
(3620)	V$TCF4_Q5_01	TCF-4	39901888 (+)	1.000	0.974	acCAAAGaat
	V$TCF7L2_07	TCF-4	39901944 (+)	1.000	0.969	tcataTCAAAggaa
	V$TCF4_06	TCF-4	39901945 (-)	1.000	0.977	cataTCAAAggaa
	V$TCF4_Q5_02	TCF-4	39901946 (+)	1.000	0.998	atatCAAAGg
	V$TCF4_01	TCF-4	39901947 (-)	1.000	0.979	taTCAAAgg
	V$TCF7L2_06	TCF-4	39901947 (-)	1.000	0.958	tatCAAAGg
	V$TCF4_Q5_01	TCF-4	39901948 (+)	1.000	0.998	atCAAAGgaa
	V$TCF4_Q5	TCF-4	39901948 (-)	1.000	0.992	atCAAAGg
	V$TCF4_04	TCF4	39901964 (+)	1.000	0.999	caCACCTgga
	V$TCF7L2_07	TCF-4	39902019 (+)	1.000	0.932	gtattTCAAAgaaa
	V$TCF4_06	TCF-4	39902020 (-)	1.000	0.944	tattTCAAAgaaa
	V$TCF4_Q5_02	TCF-4	39902021 (+)	1.000	0.989	atttCAAAGa
	V$TCF7L2_06	TCF-4	39902022 (-)	1.000	0.922	tttCAAAGa
	V$TCF4_Q5	TCF-4	39902023 (-)	1.000	0.975	ttCAAAGa
	V$TCF4_Q5_01	TCF-4	39902023 (+)	1.000	0.956	ttCAAAGaaa
	V$TCF4_06	TCF-4	39902297 (+)	1.000	0.888	gtcaTTTGAtttt
IDO2	V$TCF4_01	TCF-4	39934311 (-)	1.000	0.950	gaTCAAAac
(169355)	V$TCF4_06	TCF-4	39934526 (+)	1.000	0.891	tcctTTTGAactt
	V$TCF4_Q5_01	TCF-4	39934533 (-)	1.000	0.977	gaaCTTTGct
	V$TCF4_Q5_02	TCF-4	39934535 (-)	1.000	0.962	aCTTTGctat
	V$TCF4_04	TCF4	39934635 (-)	1.000	0.969	tggAGGTGgg
	V$TCF4_04	TCF4	39934758 (-)	1.000	0.964	ctgAGGTGgt
	V$TCF4_Q5_02	TCF-4	39934813 (+)	1.000	0.953	aatgCAAAGg
AADC	V$TCF4_06	TCF-4	39934526 (+)	1.000	0.891	tcctTTTGAactt
(1644)	V$TCF4_Q5_01	TCF-4	39934533 (-)	1.000	0.977	gaaCTTTGct
	V$TCF4_Q5_02	TCF-4	39934535 (-)	1.000	0.962	aCTTTGctat
	V$TCF4_04	TCF4	39934635 (-)	1.000	0.969	tggAGGTGgg
	V$TCF4_04	TCF4	39934758 (-)	1.000	0.964	ctgAGGTGgt
	V$TCF4_Q5_02	TCF-4	39934813 (+)	1.000	0.953	aatgCAAAGg
MAO-A	V$TCF4_04	TCF4	43655790 (+)	1.000	0.971	agCACCTcct
(4128)	V$TCF4_04	TCF4	43655882 (+)	1.000	0.968	acCACCTcta
	V$TCF4_04	TCF4	43655921 (+)	1.000	0.971	agCACCTcct
	V$TCF4_04	TCF4	43655924 (+)	0.878	0.878	acCTCCTgca
	V$TCF4_04	TCF4	43655973 (+)	1.000	0.964	ccCACCTcag
	V$TCF4_04	TCF4	43656014 (+)	0.878	0.878	acCTCCTgca

MAO-B	V$TCF4_04	TCF4	43882587 (+)	1.000	0.947	gtCACCTagg
(4129)	V$TCF7L2_07	TCF-4	43882595 (+)	1.000	0.958	ggactTCAAAgagt
	V$TCF4_06	TCF-4	43882596 (-)	1.000	0.963	gactTCAAAgagt
	V$TCF4_Q5_02	TCF-4	43882597 (+)	1.000	0.990	acttCAAAGa
	V$TCF7L2_06	TCF-4	43882598 (-)	1.000	0.964	cttCAAAGa
	V$TCF4_Q5	TCF-4	43882599 (-)	1.000	0.975	ttCAAAGa
	V$TCF4_Q5_01	TCF-4	43882599 (+)	1.000	0.955	ttCAAAGagt
ALDH2	V$TCF4_04	TCF4	111766423 (-)	1.000	0.992	gccAGGTGgt
(217)	V$TCF4_04	TCF4	111766539 (+)	1.000	0.962	gtCACCTcgt
	V$TCF4_04	TCF4	111766557 (+)	1.000	0.964	ttCACCTccg
	V$TCF4_04	TCF4	111766601 (+)	1.000	0.989	ttCACCTgga
	V$TCF7L2_07	TCF-4	111766659 (+)	1.000	0.895	tggggTCAAAggca
	V$TCF4_06	TCF-4	111766660 (-)	1.000	0.892	ggggTCAAAggca
	V$TCF4_Q5_02	TCF-4	111766661 (+)	1.000	0.974	gggtCAAAGg
	V$TCF7L2_06	TCF-4	111766662 (-)	1.000	0.936	ggtCAAAGg
	V$TCF4_Q5	TCF-4	111766663 (-)	1.000	0.975	gtCAAAGg
AANAT	V$TCF4_04	TCF4	76467549 (-)	1.000	0.968	gagAGGTGgg
(15)	V$TCF4_04	TCF4	76467727 (-)	1.000	0.954	ggtAGGTGgg
	V$TCF4_04	TCF4	76467742 (+)	0.878	0.883	caCTCCTggc
	V$TCF4_04	TCF4	76467766 (-)	0.878	0.881	aacAGGAGct
	V$TCF4_04	TCF4	76467840 (-)	1.000	0.959	ggaAGGTGga
ASMT	V$TCF4_Q5_01	TCF-4	1615052 (-)	1.000	0.978	cgtCTTTGtt
(438)	V$TCF4_Q5_02	TCF-4	1615054 (-)	1.000	0.974	tCTTTGtttg
	V$TCF4_04	TCF4	1615120 (+)	1.000	0.958	ccCACCTtgc
TCF7L2	V$TCF4_Q5_01	TCF-4	112950322 (-)	1.000	0.955	cgcCTTTGaa
(6934)	V$TCF7L2_07	TCF-4	112950322 (-)	1.000	0.962	cgccTTTGAactga
	V$TCF4_06	TCF-4	112950322 (+)	1.000	0.964	cgccTTTGAactg
	V$TCF7L2_06	TCF-4	112950324 (+)	1.000	0.965	cCTTTGaac
	V$TCF4_Q5_02	TCF-4	112950324 (-)	1.000	0.988	cCTTTGaact
	V$TCF4_Q5	TCF-4	112950324 (+)	1.000	1.000	cCTTTGaa
	V$TCF4_04	TCF4	112950381 (+)	1.000	0.971	agCACCTcct
	V$TCF4_04	TCF4	112950384 (+)	0.878	0.878	acCTCCTgta
	V$TCF4_Q5_01	TCF-4	112950409 (-)	1.000	0.980	cccCTTTGct
	V$TCF4_Q5_02	TCF-4	112950411 (-)	1.000	0.962	cCTTTGctct

Supplementary table 4: Summary of position weighted matrices for TCF7L2 which have been matched to promoter binding sites. Matching matrices as depicted in supplementary figure are summarized with respective chromosomal positions, sequences, and scores, described in detail in the TRANSFAC database (release2018.2).

B) Supplemental figures

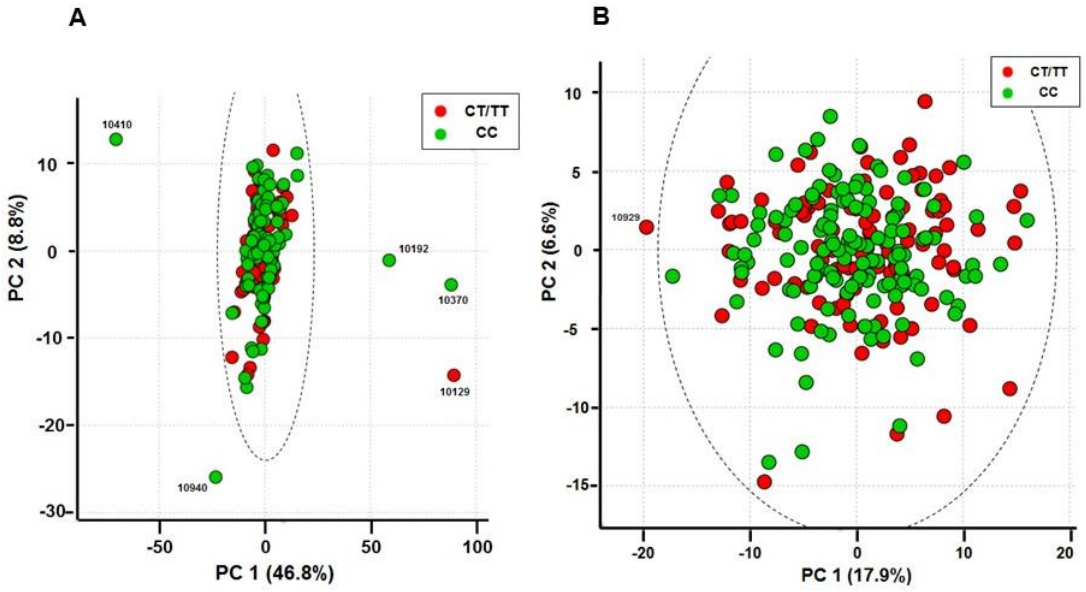

Supplementary figure 1: PCA-outlier detection.
The plot illustrates principal components analysis (PCA) between principal component (PC) 1 and 2 in the discovery (A) and validation study (B). Data were generated from MS normalised data of serum samples from patients carrying the TT/CT and the CC genotype at TCF7L2 SNP rs7903146. Explained variances are shown in brackets. Outliers are identified by distance to origin and highlighted by sample names.

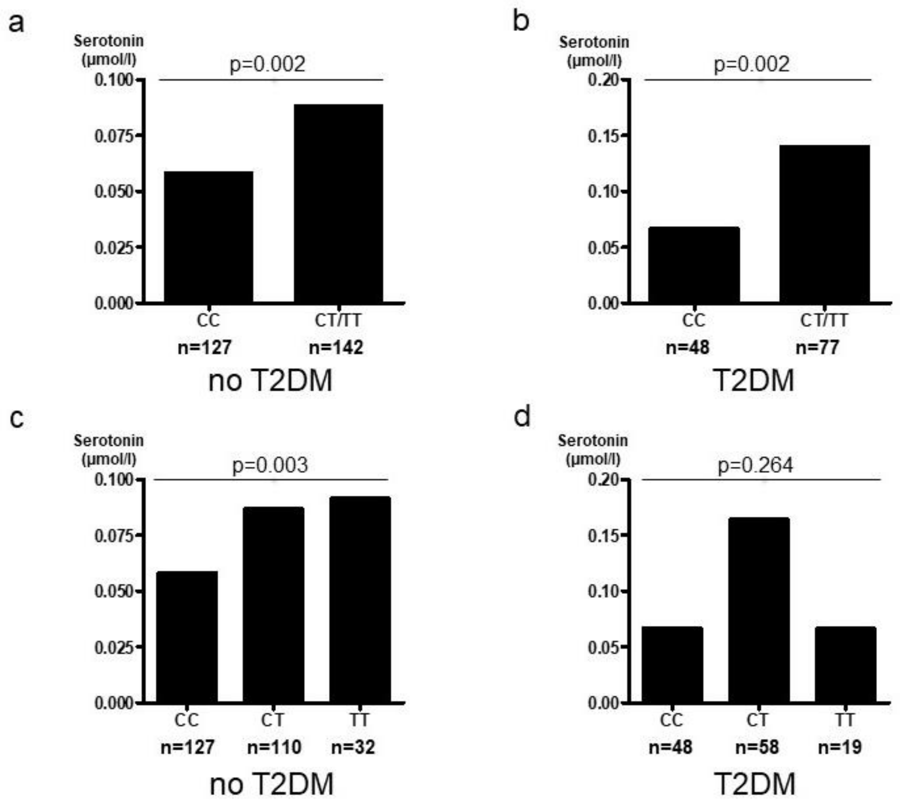

Supplementary figure 2: Serotonin concentration in subjects with respect to their rs7903146 genotype.

The figure indicates the mean serotonin concentration (µmol/l) in subjects (a, b) with either the risk allele (CT/TT) or lacking the risk allele (CC) and in subjects (c, d) carrying the CC, CT, or TT allele, being (b, d) affected by type 2 diabetes mellitus (T2DM) or being (a, c) not affected by T2DM. P-values are calculated according to Mann-Whitney-U and Jonckheere-Terpstra-test for trend respectively.

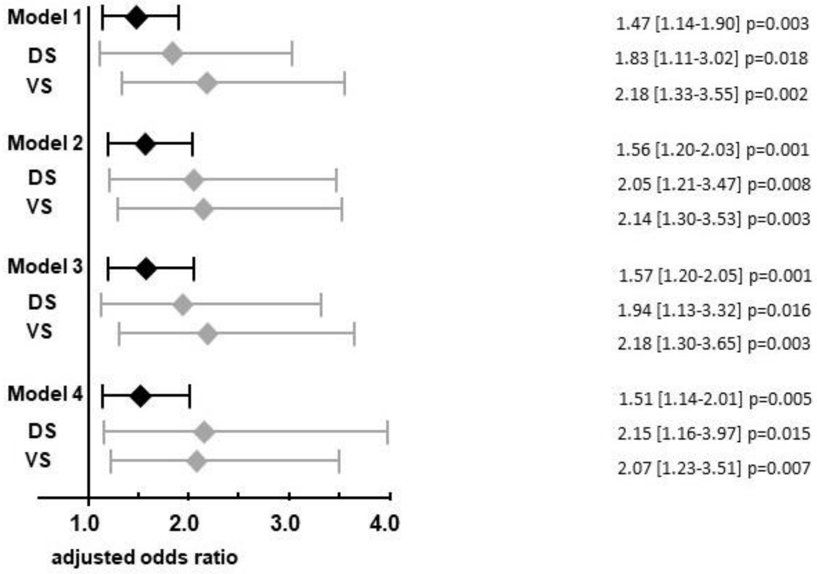

Supplementary figure 3: Association between TCF7L2 rs79303146 genotype and serotonin in patients without diabetes.

The forest plot depicts the odds ratios (OR) and 95% CI derived from binary logistic regression analysis for the association between TCF7L2 rs79303146 genotype and serum serotonin concentration as a continuous variable with stepwise adjustment in patients without type 2 diabetes. Adjustment model 1, a univariate model, represents the association between the genotype and serotonin. Model 2 represents the association between the genotype and serotonin with covariates age, gender, and body mass index. Model 3 accounts for the parameters included in model 2 and in addition the hypertension status, the sig. CAD status, and the current smoking status. Model 4 accounts for the parameters included in model 3 and in addition, arginine and the phosphatidylcholin PC aa C38:6. DS denotes discovery study, VS validation study. Patients with T2DM (n=125) have been excluded.

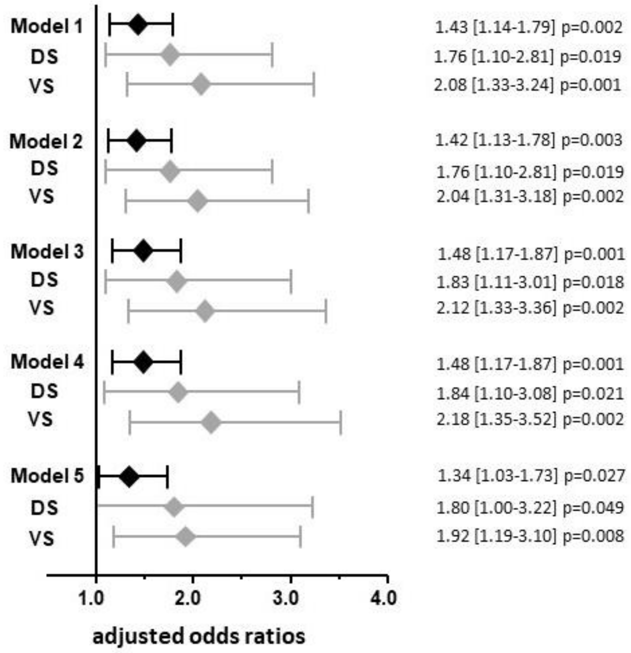

Supplementary figure 4: Association between TCF7L2 rs79303146 genotype and serotonin in patients without serotonin impacting medication.

The forest plot depicts the odds ratios (OR) and 95% CI derived from binary logistic regression analysis for the association between TCF7L2 rs79303146 genotype and serum serotonin concentration as a continuous variable with stepwise adjustment in patients without type 2 diabetes. Adjustment model 1, a univariate model, represents the association between the genotype and serotonin. Model 2 represents the association between the genotype and serotonin with T2DM as covariate. Model 3 accounts for the parameters included in model 2 and in addition covariates age, gender, and body mass index. Model 4 accounts for the parameters included in model 3 and in addition the hypertension status, the sig. CAD status, and the current smoking status. Model 5 accounts for the parameters included in model 4 and in addition, arginine and the phosphatidylcholin PC aa C38:6. DS denotes discovery study, VS validation study. Patients who have received anti-depressive drugs impacting serotonin concentration (n=44) have been excluded.

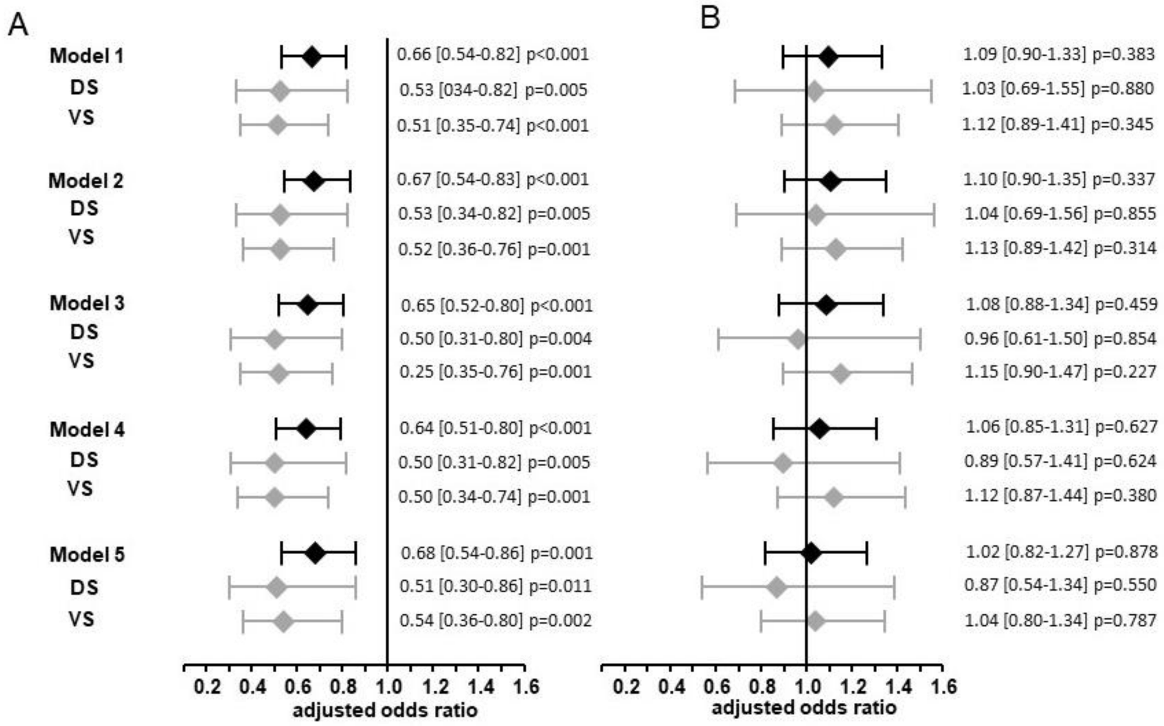

Supplementary figure 5: Association between TCF7L2 rs79303146 genotype and (A) the Trp-serotonin ratio and (B) the Trp-kynurenine ratio.

The forest plots depict the odds ratios (OR) and 95% CI derived from binary logistic regression analysis for the association between TCF7L2 rs79303146 genotype and the respective ratios as continuous variables with stepwise adjustment. Adjustment model 1, a univariate model, represents the association between the genotype and serotonin. Model 2 represents the association between the genotype and serotonin with the T2DM status as covariate. Model 3 includes the parameters included in model 2 and in addition the covariates age, gender, and body mass index. Model 4 includes the parameters included in model 3 and in addition the hypertension status, the sig. CAD status, and the current smoking status. Model 5 includes the parameters included in model 4 and in addition, arginine and the phosphatidylcholin PC aa C38:6. DS denotes discovery study, VS validation study.

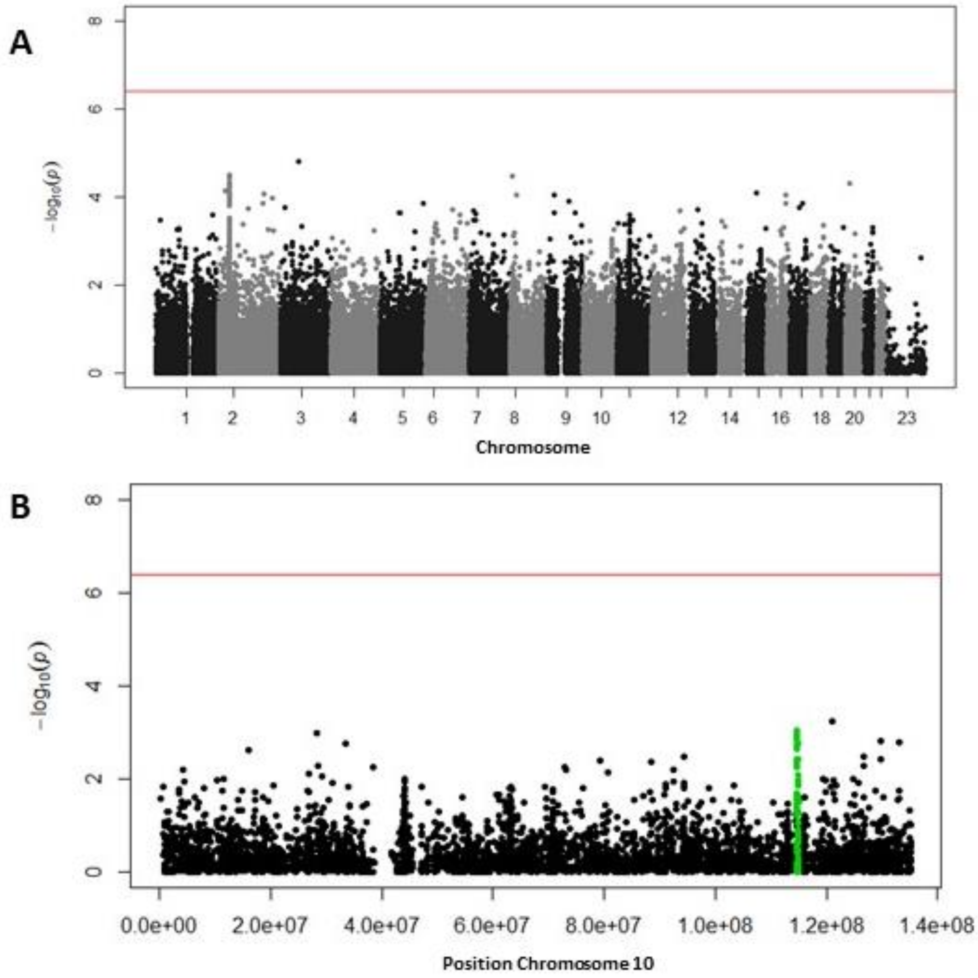

Supplementary figure 6: Manhattan plot of genome wide (A) and chromosome wide (B) association study of serotonin concentration.

SNPs were characterized using the Illumina Cardio-Metabo Chip. After frequency and genotyping pruning, there were 130,909 SNPs left. The red line indicates genome-wide significant associations (3.81 E-7). TCF7L2 genomic region is highlighted in green.

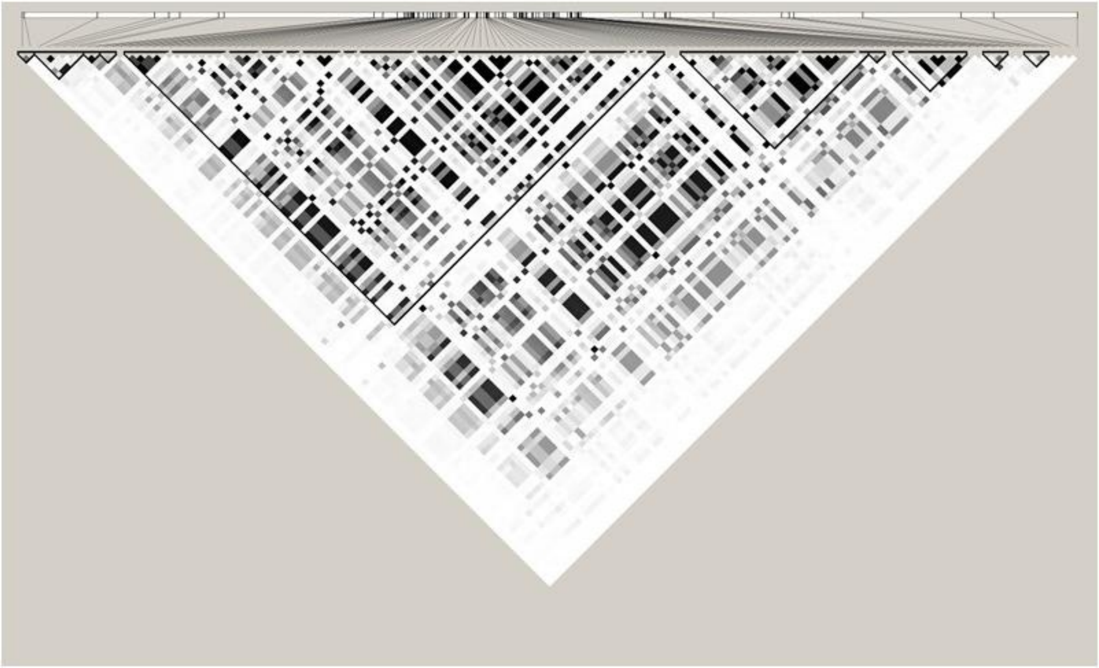

Supplementary figure 7: LD plot.
The plot indicates the linkeage disequilibrium (LD) of 130 polymorphisms between position 112.910 kb and 113.278 kb (GRCh38.7) on chromosome 10. Nineteen tagging SNPs were needed to cover the complete genomic region at $r^2 \geq 0.9$.

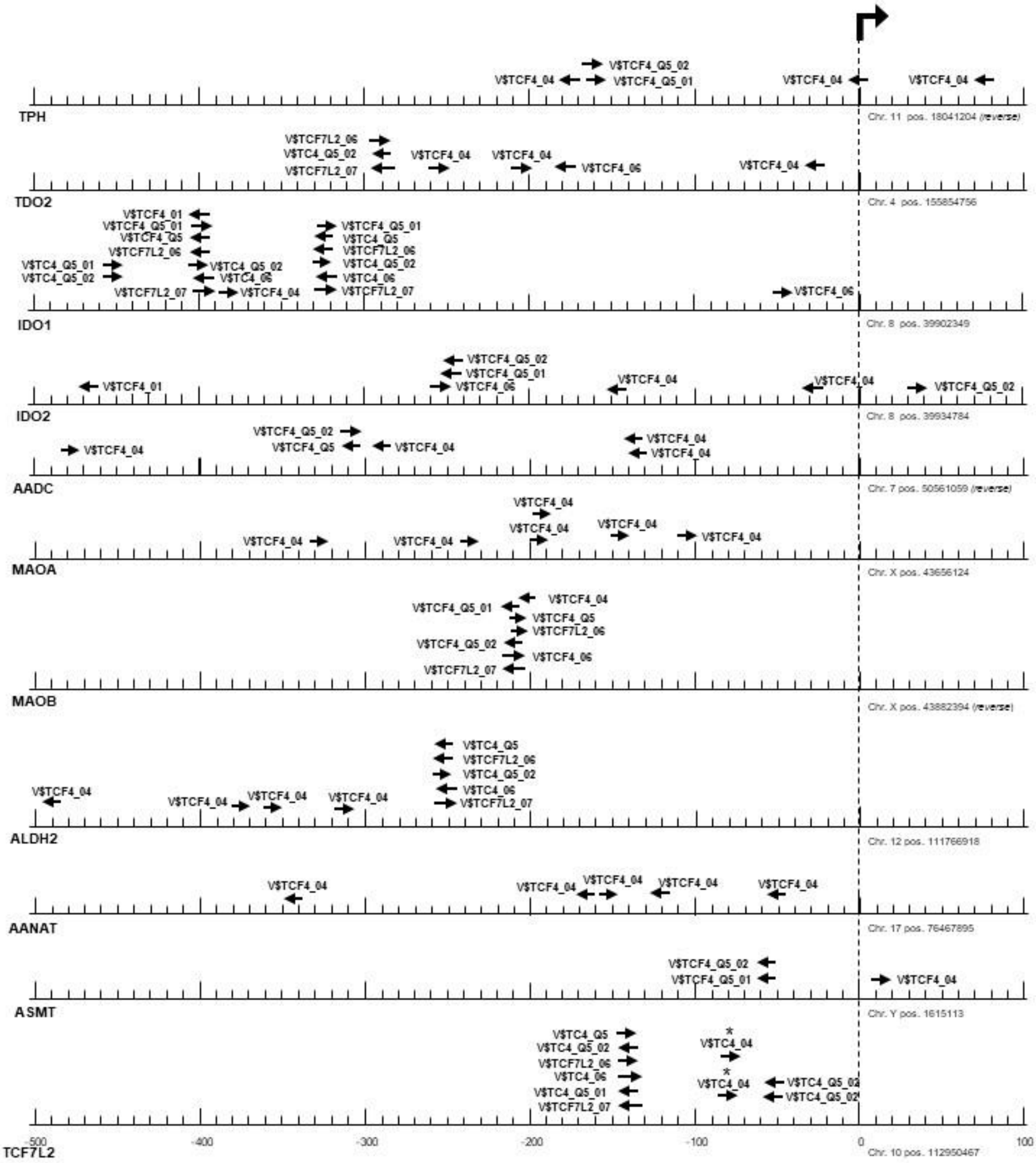

Supplementary figure 8: Binding site prediction for transcription factor TCF7L2 (TCF4) within promoter regions of TPH, TDO2, IDO1, IDO2, AADC, MAO-A, MAO-B, ALDH2, AANAT, ASMT, and TCF7-L2.

Matching matrices (arrows) are shown within the proximal promoters representing transcription factor binding sites. The result was filtered according to the minimization of the sum of false positive and false negative results. Experimental validated binding is marked by asterisks.

C) Supplemental methods section

Study subjects

All patients were selected from a coronary angiographically characterized cohort. These patients have been consecutively recruited for angiography for evaluation of established or suspected CAD in our hospital (Academic Teaching Hospital Feldkirch). Coronary angiography was performed with the Judkin's technique and the severity was assessed by visual inspection as described previously [1]. Coronary artery stenosis with lumen narrowing ≥50% was referred to as significant CAD. Patients with acute coronary syndromes or those with type 1 diabetes were excluded from the study. Type 2 diabetes mellitus (T2DM) was diagnosed according to American Diabetes Association (ADA) guidelines [2] in the presence of any of: fasting plasma glucose ≥126 mg/dl (7.0 mmol/l), plasma glucose ≥200 mg/dl (11.1 mmol/l) after oral glucose tolerance test (OGTT), or haemoglobin A1c (HbA1c) ≥6.5% (47.5 mmol/mol), and anamnestic known diabetes. BMI was calculated as body weight (kg) / height (m^2), and BMI≥30 was regarded as obesity. Current smoking status was applied for patients currently smoking or having quit smoking <1month prior to the study. A positive alcohol consumption status was applied in case of any alcohol consume.

Laboratory analyses

Basic clinical measurements and laboratory analyses were performed at the Medical Central Laboratories Feldkirch. Venous blood samples were collected in the morning after an overnight fast of 12 h, centrifuged immediately, and used for basic laboratory tests. Basic laboratory measurements were immediately performed from fresh serum samples. Aliquots of samples were frozen and stored at -80°C for metabolomic profiling. Plasma glucose was determined on a Cobas Integra 800® (Roche, Basel,

Switzerland) and HbA1c by high performance liquid chromatography on a Menarini-Arkray KDK HA 8140® (Arkray KDK, Japan). OGTT was performed after an oral 75g glucose challenge. The serum levels of total cholesterol, low-density lipopoprotein (LDL) cholesterol and high-density lipoprotein (HDL) cholesterol were determined by using enzymatic hydrolysis and precipitation techniques on a Hitachi-Analyzer 717 or 911 (QuantolipLDL, QuantolipHDL; Roche, Basel, Switzerland). Serum creatinine were measured using the modified Jaffe method (Creatinine Jaffe Gen.2 Assay, Roche, Switzerland) to estimate the glomerular filtration (eGFR) according to the quadratic Mayo Clinic equation [3].

Cardio-Metabo chip and genotyping

Patient samples were excluded if they had genotyping failure for more than 5% of the sites or if they were identified as duplicates. The chip comprises a total of 196,725 SNPs. Frequency and genotyping pruning led to the exclusion of SNPs if they failed to be genotyped in more than 5% of the study population, if they were not in Hardy-Weinberg equilibrium among samples (critical p-value=0.0001), or had a minor allele frequency less than 20%. With these settings, we did not observe significant genomic inflation (λ= 1.012). Genome-wide significance was 3.81E-7.

According to this frequency and genotyping pruning, the genomic region of TCF7L2 gene, located on chromosome 10 position 112.910 kb - 113.278 kb (GRCh38), is covered by 130 SNPs. These SNPs were further captured by 19 tagging SNPs applying a r^2 threshold of 0.9 for the strength of linkage disequilibrium using the program Haploview version 4.2 (https://www.broadinstitute.org/haploview/haploview).

Transcription factor analysis

Transcription factor analysis was done in silico applying the Match search algorithm [4] in combination with the professional version of TRANSFAC database (release 2018.2), implemented in the geneXplain platform (http://genexplain.com/genexplain-platform/; GeneXplain, Wolfenbüttel, Germany) as described previously in detail [5]. In short, high quality position weighted matrices predicting TCF7L2 (TCF4) binding sites in human were combined in a user-defined profile. Match algorithm was used with cut offs for this profile to minimize the sum of false negative and false positive results. Prediction analysis was done on proximal promoter regions (-500 to +100 bp with respect to the transcription start) of genes involved in serotonin metabolism and of TCF7L2.

D) Supplemental references

1. Drexel H, Amann FW, Beran J, Rentsch K, Candinas R, Muntwyler J, Luethy A, Gasser T, Follath F. Plasma triglycerides and three lipoprotein cholesterol fractions are independent predictors of the extent of coronary atherosclerosis. (1994) Circulation 90:2230–2235.

2. American Diabetes Association. Standards of Medical Care in Diabetes—2015. (2015) Diabetes Care 38:S1–S93.

3. Rule AD, Larson TS, Bergstralh EJ, Slezak JM, Jacobsen SJ, Cosio FG. Using serum creatinine to estimate glomerular filtration rate: accuracy in good health and in chronic kidney disease. (2004) Ann. Intern. Med. 141:929–37.

4. Kel AE, Gössling E, Reuter I, Cheremushkin E, Kel-Margoulis O V, Wingender E. MATCH: A tool for searching transcription factor binding sites in DNA sequences. (2003) Nucleic Acids Res. 31:3576–9.

5. Leiherer A, Geiger K, Muendlein A, Drexel H. Hypoxia induces a HIF-1α dependent signaling cascade to make a complex metabolic switch in SGBS-adipocytes. (2014) Mol. Cell. Endocrinol. 383:21–31.
